# The Antibacterial and Anti-biofilm Traits of the Novel BMAP-27-Melittin Conjugated Peptide Nanoparticle Against *Streptococcus mutans: *Clinical Isolates from Oral Cavity

**DOI:** 10.30699/IJP.2022.547555.2718

**Published:** 2022-08-13

**Authors:** Mohammad Pourahmadi, Kimia Pourahmadi, Farzan Modaresi, Shekoufeh Atashpour, Azita Azad, Alireza Ranjbaran, Abdolmajid Ghasemian

**Affiliations:** 1 *Department of Anatomical Sciences, Jahrom University of Medical Sciences, Jahrom, Iran*; 2 *Student Research Committee, School of Dentistry, Shiraz University of Medical Sciences, Shiraz, Iran*; 3 *Departments of Microbiology, Advanced Medical Sciences and Technology, Jahrom University of Medical Sciences, Jahrom, Iran*; 4 *Department of Pharmacology, Advanced Medical Sciences and Technology, Jahrom University of Medical Sciences, Jahrom, Iran*; 5 *Oral and Dental Disease Research Center, Department of Oral & Maxillofacial Medicine, School of Dentistry, Shiraz University of Medical Sciences, Shiraz, Iran*; 6 *Noncommunicable Diseases Research Center, Fasa University of Medical Sciences, Fasa, Iran*

**Keywords:** Antimicrobial Peptide, Biofilms, BMAP-27, Melittin, Nanoparticles, Streptococcus mutans

## Abstract

**Background & Objective:**

The spread and development of drug-resistant bacterial strains has prompted the hunt for novel antibacterial polypeptides undergoing conformational changes to confer rapid bactericidal effects. The aim of this study was to evaluate the effect of novel BMAP27-Melittin conjugated peptide- nanoparticle (NP) against *Streptococcus mutans *as the primary pathogen from subgingival plaques.

**Methods:**

Sixty subgingival plaque samples were collected, and 39 *S. mutans* isolates were identified*.* The BMAP27-Melittin conjugated peptide was purchased from GenScript Company, USA. Minimum Inhibitory Concentration (MIC), Minimum Bactericidal Concentration (MBC), Biofilm Inhibitory Concentration (BIC), and Biofilm Eradication Concentration (BEC) of BMAP27-Melittin-NP were calculated using the microtiter method.

**Results:**

Thirty-nine infected subjects were reported, including 24 males and 15 females (*P*=0.299). MIC, MBC, BIC, and BEC of BMAP27-Melittin–NP against *S. mutans* were 1.8, 2.9, 2.1, and 3.8μg/mL, respectively. The mean MBC, BEC, and BIC values were significantly lower among clinical isolates than *S. mutans* ATCC 35688 standard strain (*P*=0.032, 0.001, and 0.001, respectively).

**Conclusion:**

BMAP27-Melittin-NP demonstrated significant antibacterial and anti-biofilm effects against clinical isolates of *S. mutans* which can be considered a promising compound to prevent or treat dental caries and eradicate the oral infections.

## Introduction

Dental caries is one of the world's most serious health issues and the most common and costly disease. A biofilm slime coating ensubjects the tooth's surface, composed of millions of bacterial cells, salivary polymers, and food particles. The tooth's surface is enveloped with a biofilm slime layer consisting of millions of bacterial cells, salivary polymers, and food debris. This biofilm, also known as plaque, provides an excellent attachment surface for many bacterial species to colonize and expand (1). Biofilms are surface-attached groups of microbes embedded in an extracellular polymeric material matrix that cause various chronic and recurring infections. Therefore, biofilms are one of the major participants in the universal health problem causing antibiotic resistance, necessitating the development of novel approaches to combat them (2, 3).

*Streptococcus mutans (S. mutans) *has a substantial role in the etiology of dental caries due to its ability to attach to the enamel salivary pellicle and other plaque bacteria. *S. mutans* is a primitive domain of the mouth, pharynx, and intestine, a strong acid producer which cause tooth decay by developing an acidic environment (4, 5). In most subjects, the presence of *S. mutans* in tooth cavities is accompanied by caries within 6-24 months. In the presence of sucrose, acidogenic *S.mutans* can produce extracellular polysaccharides (EPS) and fructose homopolysaccharides. The ability of *S.mutans* to produce vast amounts of EPS from sucrose is a key factor in its cariogenicity (6, 7). *S.mutans* DNA has been found in a greater percentage of cardiovascular specimens than in other periodontal bacteria, suggesting involvement in a variety of cardiovascular disorders other than bacteremia and infective endocarditis (8, 9).

Over the past several decades, inappropriate antibiotic prescription and consumption have contributed to the spread and development of those multidrug- and extensively drug-resistant (MDR and XDR, respectively) bacterial strains. Nowadays, bacterial drug resistance is a major public health problem all over the world. The spread of drug-resistant strains has led to the failure to eradicate infections and limited the efficient antibiotics (10). Nanoparticles (NPs) and antimicrobial peptides have recently been considered novel compounds to combat MDR and XDR strains (11, 12).

Antimicrobial peptides (AMPs) are small molecules of wide antimicrobial activity against bacterial, fungal, parasitic, and enveloped viral agents (13). AMPs are generally 10–50 amino acids in length, exhibiting a cationic nature and forming amphipathic structures in contact with the cell membranes. AMPs have sparked considerable concern as potential antibiotic pharmaceuticals due to their appealing properties of broad-spectrum antimicrobial action and rapid killing kinetics (14, 15). However, AMPs have poor antibacterial target selectivity and confer cytotoxicity against mammalian cells, which is a drawback in their clinical application (16). Various techniques have been employed to enhance the selectivity of AMPs and improve their therapeutic index, including hybrid AMP (17).

Hybridizing two peptides of distinct physicochemical properties is an effective method to obtain novel AMPs exerting higher antibacterial activities or lower cytotoxicity against mammalian cells. Several hybrid AMPs have been developed based on the hybridization of various segments, resulting in substantial changes in biological activities and toxicity profiles compared to the primary compounds having promising insights as novel therapeutics (17, 18).

Bovine myeloid antimicrobial peptide-27 (BMAP-27) is a small compound with α-helix or β-sheet linear motifs and a linear or cyclic structure with antibacterial and antifungal traits (19). The BMAP-27 helix-hinge-helix structure and the cationic aromatic cluster of the N-terminal-helix cause the cytoplasmic membrane disruption following peptide-membrane interaction. The rapid bactericidal action and the effective depolarization of cytoplasmic membranes have highlighted that BMAP-27 functions mainly against the cellular membrane (20).

Melittin is the most crucial toxin in the European honeybee *Apis mellifera* venom, which consists of 26 amino acids and has cationic and hemolytic properties. Owing to the inclusion of a sequence of amino acids, melittin is an amphoteric peptide containing hydrophilic carboxy-terminal and cationic hydrophobic amino-terminal regions. Melittin has demonstrated interactions with biological membranes or enzymes with amphiphilic properties, potentiating it as an efficient antimicrobial and anticancer compound (21-23).

BMAP27-Melittin consists of an N-terminal fragment obtained from residues 9-20 of BMAP-27 and a C-terminal fragment from residues 2–9 of melittin. BMAP27-Melittin is made up of 21 amino acid residues and has antimicrobial activity against a wide variety of gram-positive and gram-negative bacteria at concentrations ranging from 1-7.5 M. This conjugated peptide has exhibited potential antibacterial (minimum inhibitory concentration (MIC) value of 1 μM) and antibiofilm effects against MDR strains without cytotoxicity against normal eukaryotic cells at these concentrations. Additionally, the hybrid peptide has folded into a well-defined α-helical structure, according to molecular dynamics simulations of peptide folding (24).

Peptide-nanoparticle conjugation (PNC) permits enhanced control over the structural properties of nanostructures, enabling comfortable conformational alterations of conjugates by designing NP scaffolds specifically for intended applications in biomedical research (25). The synergy between the two existing classes of materials allows for more proper control of their biological activities, overcoming the inherent shortcomings of each compound. PNCs have been developed for various applications such as drug delivery, inhibition of pathogenic biomolecular interactions, molecular imaging, and liquid biopsy (26, 27). The integration of NPs with proteins has been shown to alter protein conformation, the nanoparticle's surface properties, and colloidal stability (28). In this study, we aimed to design the novel BMAP-27-Melittin conjugate and its antimicrobial activity against *S. mutans *isolated from the oral cavity.

## Material and Methods

**Ethical Approval**

This study was approved by the Ethical Committees of Medical Research of Shiraz University of Medical Sciences with the ethical code number: IR.SUMS.DENTAL.REC.1399.119.

**Samples and Demographic Data**

In this study, the saliva samples were collected from 60 patients older than 18 years who were referred to the Shiraz Dental School's Oral and Dental Diagnosis Department (convenient non-random sampling). The participants had more than 15 teeth without the active periodontal disease (no attachment loss level more than 5mm during dental examination) or periodontal treatments in the last six months. The subjects with pregnancy, diabetes or autoimmune disease, antibiotics, or anti-inflammatory consumption during the last three months, or needing antibiotic prophylaxis for dental treatments were excluded. A questionnaire was prepared with personal information (including age, sex, underlying disease, rate and method of toothbrush use, oral lesion or wound status and date of sampling for each patient). The participant’s written consent was taken before initiation of the study. After supragingival scaling and isolation with a rubber dam, samples were taken by one of the authors as described by Gomes* et al.* (29). The teeth and the adjacent field were decontaminated with a 2.5% sodium hypochlorite for 30 s and then inactivated with 5% sodium thiosulfate. As the previous restorations were removed and the access cavities were available, the pulp chambers were disinfected using 5.25% sodium hypochlorite, and the obturation materials were removed using ProTaper nickel-titanium rotary instruments SX-F2 (WNT, India) under irrigation with sterile saline. The samples were collected, inserting two sterile paper points into the canal's working length and keeping them in place for 60 s. The debris on the paper points were transferred into sterile 2 mL Eppendorf tubes containing Gotenberg agar III transport medium and evaluated immediately within 2 hrs. After shaking the samples for 60 s (Vortex, Scientific Industries Inc., Springfield, MA), 1 mL of each sample was used for culture, and the other 1 mL was kept at −20°C for polymerase chain reaction (PCR) procedures (29). 

**Bacterial Identification using PCR Technique**

The total genomic DNA was extracted using a QIAGEN DNA extraction kit according to the protocol. The DNA quantity was measured using a spectrophotometer at 260/280nm. The PCR technique was employed to amplify the 16S rRNA gene (433 bp size) using Forward: 5'-GGCACCACAACAT-TGGGAAGCTCAGTT-3' and Reverse: 5'-GGAATGGCCGCTAAGTCAACAGGAT-3' primers. In a total volume of 25µL, Master Mix (QIAGEN) (10µL), ddH2O (13µL), and each primer (1µL) were mixed. The *S. mutans* PCR reaction conditions included initial denaturation at 94°C for 5 minutes, followed by 30 cycles of denaturation at 94°C for 45 seconds, annealing at 60°C for 45 seconds, extension at 72°C for 45 seconds, and final extension for 10 minutes. In addition, *S. mutans* ATCC 35688 standard strain was used as the control of the test. 

**BMAP27-Melittin Conjugated Peptide Preparation**

BMAP27-Melittin conjugated peptide with the sequences of WGKVLIVIKHKLKFKLKS was purchased from GenScript Company, USA. The molecular weight of the peptide was 2165.7, and its HPLC purity was ≥90.0%. The peptide was soluble in ddH_2_O and DMSO but non-soluble in 1X Dulbecco's Phosphate Buffered Saline * (pH 7.1±0.1) and then stored at -20°C.

**Nanoparticle Suspension Preparation**

The nanoparticle powder (10mg) was dissolved in 200 mL of double-distilled water (ddH_2_O) using sonication (inside an ice bucket) at 90W for 21 minutes. Then the sonicated solution was filtered using a 0.2 μM size filter, and the final concentration of NPs was determined by spectrophotometer. The NP antimicrobial effects were tested with the BMAP27-Melittin at various concentrations. However, no conjugation between the NP and the BMAP27-Melittin was conducted. 

**MIC & MBC Determination**

Minimum inhibitory concentration (MIC) of BMAP27-Melittin-nanoparticle against *S. mutans* was determined using the broth microdilution method according to the clinical and laboratory standards institute (CLSI) antimicrobial susceptibility testing guidelines for bacteria that grow aerobically. A serial dilution of BMAP27-Melittin ranging 1-100 µg/mL and nanoparticle (0.125-64 µg/mL) was prepared in Müller-Hinton broth medium (MHB, Merk) (1mL). In the next step, 1 mL of bacterial suspension (1 x 10^6^) was added to each tube. The tubes were incubated at 37°C for 24 h. The lowest concentration that inhibited bacterial growth was considered the MIC. To determine MBC, 100 μL of each dilution without growth was cultured onto the Müller-Hinton agar (MHA, Merk) medium surface. The suspension was incubated at 37°C for 24 h. The colonies were counted, and the lowest concentration, killing 99.9% of isolates, was determined as the MBC. 

**Biofilm Preparation**

*S.* *mutans* isolates suspension (equal to the 0.5 McFarland standard solution (30)) were cultured in polystyrene flat-bottomed microtiter plates containing trypticase soy broth (TSB) plus 1% sucrose. After 24h of incubation at 37°C and 5% CO_2_, the planktonic-phase cells were gently removed, and the wells were washed using PBS. Next, 200 μL of the BMAP27-Melittin- NPs dilutions (in TSB plus 1% sucrose), and TSB plus 1% sucrose (control) were added to the wells. The plates were incubated at 37°C with 5% CO_2_ for 24 h. The absorbance rate at OD600 was measured at time 0 and after incubation for 24 h by spectrophotometry using an ELISA reader. 

**BIC & BEC Determination**

The biofilm inhibitory concentration (BIC) was determined as the lowest concentration where no bacterial biofilm was formed compared to the initial value. To determine the biofilm eradication concentration (BEC), samples of biofilms from the bottom of these wells were scraped by a metal loop. They were spread on TSA plates and incubated at 37°C and 5% CO_2_ for 48hour. The BEC value was determined as the lowest concentration at which no bacterial growth occurred on the TSA media surface. 

**Statistical Analysis**

Statistical analysis was carried out using SPSS 21 (SPSS Inc., Chicago, IL., USA), applying One-way analysis of variance (ANOVA) followed by the Duncan post hoc test. Statistical P-value less than 0.05 was considered significant**.**

## Results

**Demographic Data and Bacterial Isolates**

**Fig. 1:**
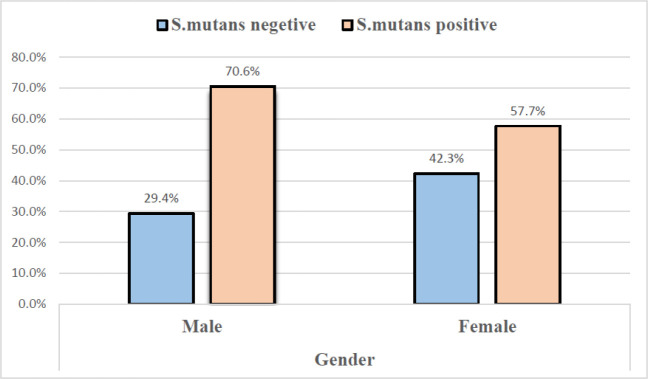
The relationship between gender and the presence of the *S. mutans*

**Fig. 2:**
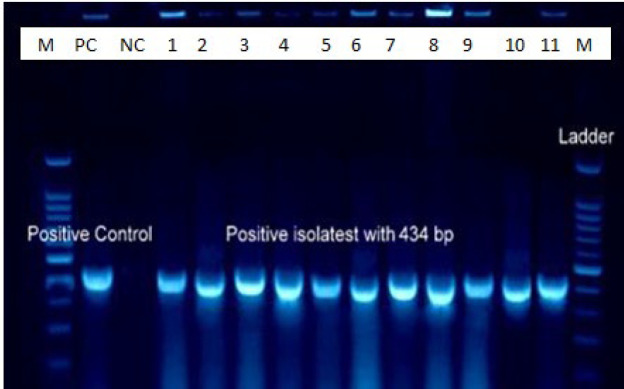
Electrophoresis of *S. mutans* 16SrRNA gene product (433 bp) on 1% agarose gel; wells: M: 100 bp DNA marker, PC: positive control, NC: negative control, 1-11: positive samples

**Fig. 3:**
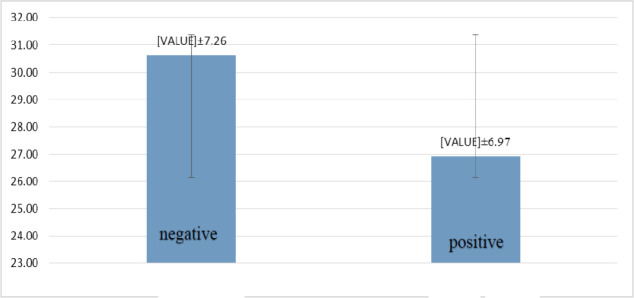
The mean age of *S. mutans*-positive and negative subjects

Gender Frequency and the mean age of the total subjects (60) included 34 males (56.7%) and 26 females (43.3%). The participants' ages ranged from 17-45 years, with a mean of 28.21±7.23. All samples were subjected to conventional PCR for *S. mutans* identification. *S. mutans* amplified in the saliva of 39 subjects (65%), including 24 men and 15 women, with no statistical differences (*P*=0.299) (Figures 1 & 2). The mean age of positive subjects (26.92±7.26) was lower than negative subjects (30.62 ±7.26), but there was no statistical difference (*P*=0.058) (Figure 3).

**BMAP27-Melittin-NPs Characterization**

**Fig. 4.**
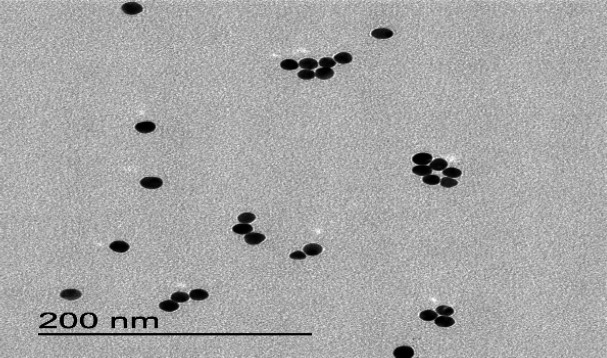
BMAP27-Melittin-NPs TEM image (30-40 nm size): JEOL Jem 1011 Transmission Electron Microscope. Mass concentration: Freeze Dryer Christ Alpha 1-4 LSC. Zeta Potential: Malvern Zetasizer Nano ZS9. UV-Vis: Spectrophotometer Nanodrop 2000c

BMAP27-Melittin-NPs were used with the aim of their antimicrobial activity estimation against *S. mutans*. Using transmission electron microscopy (TEM), NPs' average size included 30-40 nm with spherical form (Figure 4).

**Fig. 5:**
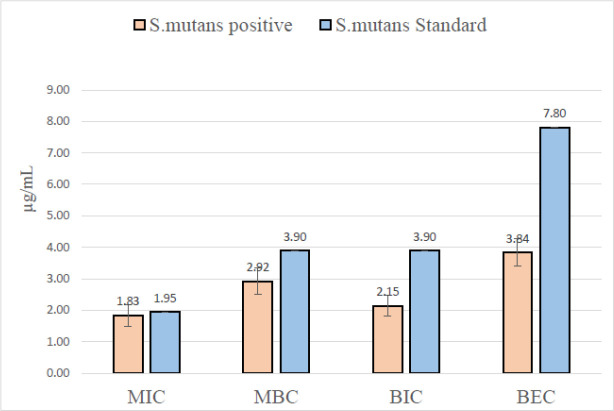
MIC, MBC, BIC and BEC value of BMAP27-Melittin conjugated peptide NPs against *S. mutans*

**Antimicrobial and Anti-biofilm Effects of BMAP27-Melittin -NPs**

For determining the antibacterial and anti-biofilm activity of BMAP27-Melittin-NPs against *S. mutans*, the MIC, MBC, BIC, and BEC included 1.8, 2.9, 2.1, and 3.8 μg/mL, respectively. The MIC value was higher against clinical isolates than the standard strain (control) without a significant statistical difference (*P*=0.732). However, the mean MBC, BEC, and BIC values against clinical isolates were significantly lower than *S. mutans* standard strain, with an average of 0.48-7.80 μg/mL in all samples (*P*=0.032, 0.001 and 0.001, respectively) (Table 1 and Figure 5). 

**Table 1:** The MIC, MBC, BIC, and BEC values of BMAP27-Melittin-NPs against *S. mutans*

Subject No.	MIC ( µg/mL)	MBC ( µg/mL)	BIC	BEC	Gender	Age
1	1.95	3.9	1.95	3.9	M	**23**
2	0.97	0.97	1.95	3.9	M	**22**
3	0.48	0.97	0.48	0.48	M	**19**
4	0.24	0.48	0.48	0.97	M	**17**
5	0.24	0.48	0.48	0.97	F	**28**
6	0.97	0.97	1.95	3.9	M	**41**
7	3.9	7.8	3.9	7.8	F	**30**
8	7.8	7.8	7.8	7.8	M	**21**
9	0.24	0.48	0.48	0.97	F	**26**
10	1.95	3.9	3.9	7.8	F	**33**
11	1.95	3.9	3.9	7.8	M	**37**
12	0.97	1.95	1.95	3.9	M	**18**
13	0.24	0.48	0.48	0.97	M	**25**
14	0.48	0.97	0.97	1.95	F	**31**
15	3.9	3.9	1.95	3.9	F	**33**
16	7.8	7.8	3.9	7.8	F	**27**
17	3.9	7.8	3.9	7.8	M	**25**
18	1.95	3.9	0.97	1.95	M	**20**
19	1.95	3.9	1.95	7.8	F	**18**
20	0.97	1.95	0.97	1.95	F	**17**
21	0.48	0.97	0.97	1.95	M	**38**
22	1.95	3.9	3.9	7.8	M	**26**
23	0.97	1.95	1.95	3.9	M	**27**
24	0.97	1.95	0.97	1.95	F	**19**
25	0.48	0.97	0.97	1.95	M	**20**
26	0.24	0.48	0.48	0.97	F	**30**
27	0.24	0.48	0.48	0.97	M	**24**
28	0.48	0.97	0.48	0.97	M	**37**
29	1.95	3.9	1.95	3.9	F	**22**
30	1.95	3.9	3.9	7.8	M	**25**
31	3.9	7.8	7.8	7.8	M	**37**
32	0.24	0.48	0.48	0.97	F	**31**
33	0.48	0.97	0.97	1.95	M	**28**
34	7.8	7.8	7.8	7.8	M	**20**
35	3.9	7.8	1.95	3.9	F	**44**
36	0.97	1.95	0.97	1.95	F	**25**
37	0.97	1.95	1.95	3.9	M	**33**
38	0.48	0.97	0.97	3.9	M	**29**
39	0.24	0.48	0.48	0.97	M	**24**
Standard strain	**1.95**	**3.9**	**3.9**	**7.8**	-	-

No: number, MIC: minimum inhibitory concentration, MBC: Minimum bactericidal concentrations, BIC: biofilm inhibition concentration, BEC: biofilm eradication concentration, M: male, F: female.

## Discussion

The emergence and development of drug-resistant clinical bacterial isolates have limited antibacterial chemotherapy. Additionally, synthetic antibiotics leave various side effects on infected patients. *S. mutans* plays a substantial role (as a major etiological agent) in oral biofilms, dental decay, and endocarditis. The oral cavity is formed by hard and soft tissue surfaces, which are potentially predisposed for development of oral biofilms (2, 5). In this study, the prevalence of *S. mutans* was 65% among the working length of root canal samples. A study by Okada *et al.* revealed that *S. mutans* was detected in 61.7% of pre-school children's plaque samples (n=60) which were collected from all erupted tooth sites using a sterile toothbrush.

Additionally, 38.5 % of the infected children had incremental caries increases (31). Another study outlined a significant association between plaque levels of *S. mutans* and caries. The strongest association was found when the plaque was removed from single occlusal fissures. Seventy-one percent of the carious fissures had *S. mutans* accounting for more than 10% of the viable flora (32).

NPs have been recently shown to be a viable alternative to synthetic chemical compounds in preventing and treating diseases. Antibacterial behavior research is focused on advanced medicine, which is the selection of nontoxic materials for therapies. Furthermore, peptides are an outstanding strategy in drug design and pharmaceutical invention due to their varied chemical features, biological function, and biotechnological relevance. A wide variety of peptides and NPs compounds have been investigated to eliminate bacterial infections and biofilms. This study evaluated the inhibitory effects of BMAP27-Melittin-NPs against *S. mutans* and related biofilm. The current study demonstrated that BMAP27-Melittin-NPs have a significant inhibitory effect on *S.mutans*. 

A study by Almaaytah *et al.* regarding BMAP27-Melittin efficacy and toxicity revealed that this novel peptide had broad-spectrum antimicrobial activity in the 1–7.5 µM range against standard gram-positive and gram-negative bacterial strains. Moreover, the peptide managed to eradicate those MDR strains with significant potency and MIC values of 1 µM. In addition, we found that BMAP27-Melittin-NPs exerted significant anti-biofilm effects, while hemolytic and antiproliferative tests on eukaryotic cells proposed its low toxicity at antimicrobial concentrations (24). 

Leung* et al.* tested the effect of KSL (α-helical peptide) on the development of oral multi-species biofilms isolated from human saliva. The colony-forming units (CFU) showed that KSL effectively inhibited biofilm growth and significantly reduced the viability of biofilm cells (33). In the current study, the BIC and BEC value was higher against clinical isolates compared to the standard strain (control), highlighting the anti-biofilm traits of the BMAP27-Melittin as an α-helical peptide.

The antibiofilm activity of synthetic peptides against major bacterial pathogens involved in the periodontal plaque biofilms has been studied previously. Wang *et al.* (2015) found that a synthetic cationic AMP Nal-P-113 conferred antibacterial and antibiofilm effects against *Streptococcus gordonii*, *Fusobacterium nucleatum*, and *Porphyromonas gingivalis,* causing periodontal disease, possibly via a pores-forming mechanism in the cytoplasmic membranes. Another study showed a synthetic peptide (BAR) could also influence interactions between bacterial strains (34) as the results of our research. The synthetic peptides have opened novel avenues toward the eradication of oral infections. Moreover, Jorge *et al.* (2012) reported that antibiofilm peptides exert activity against drug-resistant gram-negative and -positive bacteria and fungi. Generally, most AMPs permeabilize the membrane of the bacterial cells, resulting in either large-scale damage or small defects that dissipate the trans-membrane potential, eventually leading to cell death (35).

Another study by Wang (2015) investigated the ability of a cationic anti-biofilm peptide 1018 to induce the killing of bacterial cells present within oral multi-species plaque biofilms. The results showed that at 10 μg/mL (6.5μm), peptide 1018 was able to significantly (*P*<0.05) prevent biofilm formation after 72 hrs. The activity of the peptide on preformed biofilms was found to be concentration dependent. Their findings were in coordination with those of the present regarding higher inhibitory effects at higher levels, despite the differences in the compounds used (36).

According to our findings, the bacteriostatic activity of BMAP27-Melittin-NP was more prominent. There was no significant difference between this compound's bactericidal and bacteriostatic activity. Among many different factors, the capping agents play an important role in BMAP27-Melittin-NP interaction with bacterial cells, influencing bacterial growth as a bio-adhesive compound.

Noticeably, in vivo effects are also essential to verify the antibacterial properties of various compounds to overcome drawbacks in terms of therapeutic effectiveness. The findings are influenced by mechanisms such as the involvement of saliva of a certain composition, dilution, chelation, and the usual oral cleaning process. One limitation of conventional peptide-binding methods is that the peptide exposure period is insufficient to ensure precise action. In the presence of biological fluids such as plasma, serum, or saliva, the antibiofilm activity of the peptides can be significantly altered compared to their behavior in non-physiological conditions. Moreover, methods for extracting, isolating, and purifying bio-active antibiofilm peptides should be improved for large-scale manufacturing. It is also worth considering that the parenteral route of the antibiofilm peptides consumption and their excretion through the kidneys is needed to be improved, and their cell cytotoxicity must be evaluated. There is a scarcity of data regarding antibacterial and anti-biofilm effects of BMAP27-Melittin and NPs. A recent study revealed that insect peptides exert antibiofilm effects against major nosocomial pathogens via non-specific mechanisms (37). extracellular vehicles (EVs) from honeybees have exhibited antibiofilm effects against *S. mutans* and *Streptococcus sanguinis* (38). Human antimicrobial peptide LL-37 has substantial antibacterial effects (39). 

## Conclusion

The BMAP27-Melittin-NP compound exerted a significant inhibitory effect against *S. mutans* and biofilms, leaving low side effects, as a promising alternative to combat *S. mutans*. However, efforts are still needed to focus on in vivo evaluation of this peptide nanoparticle for caries prevention, anti-streptococcal activities, and its interaction with oral epithelial cells and other dentifrices composition. Therefore, the BMAP27-Melittin-NP is a potential candidate for in mouth and teeth disinfection and cleaning. 

## Data Availability

The article includes the experimental and clinical data used to support this study's findings.

## Conflict of Interest

The authors declare no conflicts of interest.

## Funding

None.
